# Effects of pressure- and volume-controlled ventilation on the work of breathing in cats using a cuffed endotracheal tube

**DOI:** 10.14202/vetworld.2021.2568-2573

**Published:** 2021-09-29

**Authors:** Nutawan Niyatiwatchanchai, Naris Thengchaisri

**Affiliations:** 1 Surgery Unit, Kasetsart University Veterinary Teaching Hospital, Faculty of Veterinary Medicine, Kasetsart University, Bangkok, 10900, Thailand; 2 Department of Companion Animal Clinical Sciences, Faculty of Veterinary Medicine, Kasetsart University, Bangkok, 10900, Thailand.; 3 Tippimarn Veterinary Hospital, Chulabhorn Royal Academy, 906/1 Pong Ta Long Subdistrict, Pak Chong District, Nakhon Ratchasima, 30130, Thailand.

**Keywords:** endotracheal tube, hypotension, pressure-controlled ventilation, respiratory work, volume-controlled ventilation

## Abstract

**Background and Aim:**

Mechanical ventilation is essential for supporting patients’ respiratory function when they are under general anesthesia. For cats with limited lung capacity, the different effects of volume-controlled ventilation (VCV) and pressure-controlled ventilation (PCV) on respiratory function remain elusive. The objective of the present study was to compare the efficacy of VCV and PCV in cats under general anesthesia using a cuffed endotracheal tube (ETT).

**Materials and Methods:**

Twelve healthy cats were randomly allocated to either a VCV or PCV group. Five tidal volumes (6, 8, 10, 12, and 14 mL/kg) were randomly applied to assess the efficacy of VCV, and respiratory rates were adjusted to achieve a minute ventilation of 100 mL/kg/min. Peak inspiratory pressures (4, 5, 6, 7, and 8 mmHg) were randomly applied to assess the efficacy of PCV, and respiratory rates were adjusted to achieve a minute ventilation of 100 mL/kg/min. Blood pressure, gas leakages, and end-tidal CO_2_ were recorded from 60 trials for airway control during the use of VCV or PCV. Data were compared using Fisher’s exact test with a significance level of p<0.05.

**Results:**

Leakages did not differ between VCV (1/60 events) and PCV (0/60 events; p=0.500). Hypercapnia was identified when using VCV (6/60 events) less frequently than when using PCV (7/60 events; p=0.762), but did not reach statistical significance. Hypotension (mean arterial blood pressure <60 mmHg) occurred less frequently with VCV (0/60 events) than with PCV (9/60 events; p=0.003). Moreover, VCV provided a significantly lower work of breathing (151.10±65.40 cmH_2_O mL) compared with PCV (187.84±89.72 cmH_2_O mL; p<0.05).

**Conclusion:**

VCV in cats using a cuffed ETT causes less hypotension than PCV. It should be noted that VCV provides a more stable tidal volume compared with PCV, resulting in a more stable minute volume. Nonetheless, VCV should not be used in patients with an airway obstruction because higher peak airway pressure may occur and lead to lung injury.

## Introduction

Endotracheal tube (ETT) intubation is considered the gold standard for maintaining airway patency when administering anesthesia to small animals, including cats [1,2]. Endotracheal intubation is indicated for patients requiring general anesthesia or those with hypoxia, respiratory fatigue, or apnea [3]. Intubation with an ETT helps prevent aspiration pneumonia and provides the option to apply controlled mechanical ventilation [1,4,5]. In practice, the depth of general anesthesia is usually controlled by spontaneous respiration and mechanical controlled ventilation. The primary indications for mechanical ventilation are persistent severe hypoxemia (PaO_2_<60 mmHg), persistent severe hypercapnia (hypoventilation), and persistent excessive respiratory effort that may lead to respiratory muscle fatigue and exhaustion, and severe circulatory shock [6,7]. Mechanical ventilation is also essential in the perioperative period for the successful treatment of many surgical procedures [8]. It supports the patient’s respiratory function while under anesthesia, promotes gas exchange, supports recovery from anesthesia, stabilizes hemodynamics in the intensive care unit, and supports weaning to successful extubation [9,10]. Furthermore, intra-abdominal hypertension leads to decreased lung volume and lung compliance, and increased airway resistance, resulting in acute respiratory distress syndrome and eventually requiring mechanical ventilation [11].

At present, there are various ventilation modes of anesthetic mechanical ventilators [12]. The most commonly used modes are volume-controlled ventilation (VCV) and pressure-controlled ventilation (PCV). VCV is the most popular mode for the perioperative period [13]. VCV has been the traditional controlled ventilation mode in anesthesia; the tidal volume is predetermined and will be delivered regardless of the associated pressure required [14]. The benefits of VCV are that it is a well-known technique and has controllable minute volume [9]. In contrast to VCV, a peak inspiratory pressure (PIP) preset is used to limit the tidal volume delivered to the patient in PCV [1]. PCV is an alternative mode of ventilation and is used primarily in patients with emergency conditions and respiratory distress [15]. PCV can improve arterial oxygenation and decrease peak airway pressure [16]. For cats with limited lung capacity, the different effects of VCV and PCV on respiratory function remain elusive. Furthermore, complications related to the use of mechanical ventilators (VCV vs. PCV) in small-sized patients have not been compared.

The aims of the present study were to compare the effects of VCV and PCV at different tidal volume settings with fixed minute ventilation in cats under general anesthesia, using a cuffed ETT. We also compared the work of breathing and its components between VCV and PCV. Other parameters (blood pressure, occurrence of gas leakage, and end-tidal CO_2_ [ETCO_2_] measured during general anesthesia) were also compared between VCV and PCV.

## Materials and Methods

### Ethical approval and informed consent

This study was approved by the Kasetsart University Institutional Animal Care and Use Committee (approval number #ACKU61-VET-046) and by the Ethical Review Board of the Office of National Research Council of Thailand (NRCT license U1-07457-2561). Written consent was obtained from all cat owners, and the experiment complied with the Kasetsart University Institutional Animal Care and Use Standards.

### Study period and location

The study was conducted from June 2018 to June 2019. The study was conducted on 12 cats visiting the Kasetsart University Veterinary Teaching Hospital, Bangkok, Thailand.

### Animals

Twelve client-owned cats (11 domestic shorthair and one Scottish fold; median age [range]: 2.5 years [10 months-3 years]; seven males and five females; median weight [range]: 3.8 kg [2.7-4.5 kg]; median body condition score [range]: 3.5 [2.5-4]) enrolled in the present study were undergoing professional dental examinations [17]. Each cat’s owner provided informed consent. All cats were clinically healthy based on a physical examination. There were no abnormalities detected in their hematogram and serum biochemistry results. Cats with severe stomatitis, pharyngitis, faucitis, and glossitis were excluded from the study. The physical status of the cats was classified according to the American Society of Anesthesiologists (ASA) as ASA I or II. Food was withheld for 12 h and water for 8 h before general anesthesia was administered. The cats were equally and randomly assigned to either the VCV or PCV group. All cats were intubated with an ETT having an internal diameter of 3.5-4.0 mm (Tuoren, Henan Tuoren Medical Device Co., Ltd., China), and the tube was lubricated with Xylocaine^®^ Jelly 2% (lidocaine hydrochloride 30 g, AstraZeneca AB, Sweden). The ETT size was selected according to the hospital’s guidelines (ETT size: 3.0 mm for 3.0-3.5 kg cats; 3.5 mm for 3.5-4.0 kg cats; and 4.0 mm for 4.0-5.0 kg cats).

### Anesthesia

All cats underwent the same anesthetic protocol applied by the same veterinarian (NN). Before undergoing anesthesia, the results of a physical examination and the cat’s body temperature, heart rate, and electrocardiogram (EKG) results were recorded. An intravenous (IV) catheter was placed in a cephalic vein, and normal saline (0.9%) (NSS, General Hospital Products Public Co., Ltd., Thailand) solution was administered at a rate of 5 mL/kg/h. The cats were pre-oxygenated for 5 min before induction. Anesthesia was induced with a slow IV infusion of propofol, and the amount of propofol (Troypofol, Troikaa Pharmaceuticals Ltd., India) needed for induction in each cat was recorded. The depth of anesthesia was assessed before intubation based on the following five criteria [18]: Palpebral reflex, jaw tone, protraction of tongue, laryngoscope on tongue, and reaction of the larynx. Before inserting the ETT, Xylocaine 10% spray (lidocaine 10 mg/puff, AstraZeneca AB) was applied to desensitize the larynx. A laryngoscope was used during ETT insertion, and its cuff was inflated to 20 cm H_2_O using a pressure gage. The ETT was secured with gauze. Using capnography, the airway connector was placed between the airway device and the Y-piece of the anesthesia machine (Flow-i, Maquet Critical Care AB, Sweden). Anesthesia was maintained with a sevoflurane vaporizer (SEVO, Singapore Pharmawealth Lifesciences, Inc., Philippines) and an oxygen/air mixture (FiO_2_ targeted at 90%) at a flow of 2 L/min, using an infant circle rebreathing system. The end-tidal concentration of sevoflurane in each cat was set at 2.5% (approximately 1 minimum alveolar concentration).

### Mechanical controlled ventilation and monitoring

**Figure-1:**
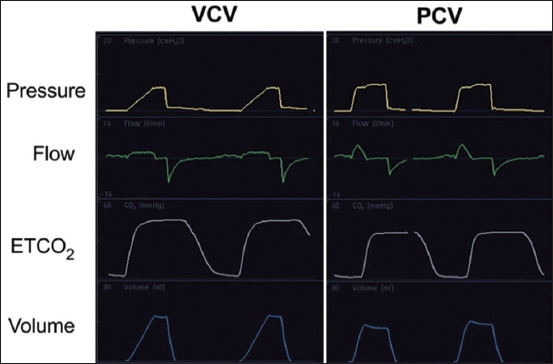
Multiple monitoring devices of pressure, flow, end-tidal CO_2_, and lung volume in cats with a cuffed endotracheal tube ventilated with volume-controlled ventilation (VCV) and pressure-controlled ventilation (PCV).

Baseline values for pulmonary and cardiovascular measurements were recorded during spontaneous ventilation after intubation. When breathing and the depth of anesthesia were stable, mechanical ventilation was initiated by the anesthesia machine (Flow-i, Maquet Critical Care AB, Sweden; Figure-1). The inspiratory-to-expiratory time ratio (I: E ratio) was set at 1:2. Ventilation of cats in the VCV group was controlled by VCV. Five inspiratory tidal volumes (VTi) (6, 8, 10, 12, and 14 mL/kg) were randomly applied during the study to assess the efficacy of VCV, and the respiratory rates (6-20 breaths/min) were adjusted to achieve a minute ventilation of 100 mL/kg/min. Ventilation of cats in the PCV group was controlled by PCV. The PIP (4, 5, 6, 7, and 8 mmHg) was randomly applied to assess the efficacy of PCV, and the respiratory rates (6-20 breaths/min) were adjusted to achieve a minute ventilation of 100 mL/kg/min. The VTi and PIP were randomly changed every 3 min in each group. Oxygen saturation (SpO_2_), heart rate, EKG, body temperature, and non-invasive blood pressure were recorded every minute by the monitoring machine (CARESCAPE Monitor B650, GE Healthcare Finland Oy, Finland). The ETCO_2_, respiratory rate, VTi, expiratory tidal volume (VTe), PIP, sevoflurane concentration, and gas leakage were monitored every minute by the anesthesia machine (Flow-i, Maquet Critical Care AB). Hypotension was defined as a mean arterial blood pressure <60 mmHg. To detect leakage, the difference between VTi and VTe was monitored. Hypercapnia was defined as ETCO_2_ >45 mmHg. Hypothermia was monitored and prevented with a water-circulating blanket (Warm Pad TP700, Soar Medical-Tech. Co., Ltd., Taiwan) placed under the cat’s body and a Bair Hugger warming blanket (Breeze, Be Hos Group Ltd., Thailand).

### Recovery

All cats were monitored for 1 h after extubation for upper respiratory airway discomfort, including stridor, coughing, retching, and hoarse voice. After full recovery from the general anesthesia, the cats were returned to their owners. The owners were instructed to record any abnormal signs in the first 24 h at home.

### Statistical analysis

STATA 12 (StataCorp, College Station, TX, USA) was used to estimate the required sample size using a *t*-test for paired samples to detect a difference in hypercapnia between VCV and PCV, using an alpha value of 0.05 (two-tailed test), a beta value of 0.8, and an effect size of 5 mmHg. All data were tested for normality using a Shapiro–Wilk test. All parametric data, including dosage of propofol and static respiratory measurements of the cats in the VCV and PCV groups, were analyzed using a paired *t*-test. A non-parametric Wilcoxon signed-rank test was used to compare the difference in the respiratory work between PCV and VCV. The association of leakage and hypercapnia was determined using Fisher’s exact test. The significance level was set at p<0.05.

## Results

**Figure-2:**
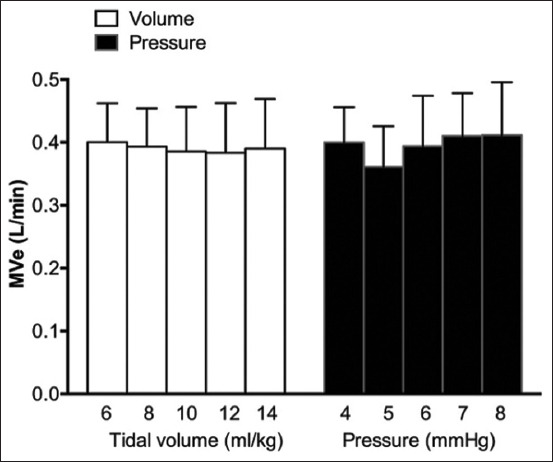
Comparison of minute ventilation in cats with a cuffed endotracheal tube ventilated with volume-controlled ventilation (VCV) and pressure-controlled ventilation (PCV).

For each tidal volume and inspiratory pressure, respiratory rates were adjusted in each cat to allow a minute ventilation of 100 mL/kg/min, and there was no significant difference in respiratory minute volume between the VCV and PCV groups (p>0.05; Figure-2). Airway leakage (>20% of the baseline tidal volume) was compared between the VCV and PCV groups (Table-1). There was no significant difference in the number of leakages between the VCV group (1/60 events) and PCV group (0/60 events; p=0.50).

**Table 1:** Airway leakage and hypercapnia identified in cats undergoing volume-controlled ventilation and pressure-controlled ventilation.

Volume-controlled ventilation						
Tidal volume (mL/kg)	6	8	10	12	14	Total
Leak>20% of baseline (no. of cats)	0	0	0	0	1	1
Hypercapnia (CO_2_>45 mmHg) (no. of cats)	3	2	1	0	0	6

**Pressure-controlled ventilation**						

Peak inspiratory pressure (cmH_2_O)	4	5	6	7	8	Total
Leak>20% of baseline (no. of cats)	0	0	0	0	0	0
Hypercapnia (CO_2_>45 mmHg) (no. of cats)	2	2	1	1	1	7

The occurrence of hypercapnia (ETCO_2_ > 45 mmHg) was compared between the VCV and PCV groups. Hypercapnia was identified when using VCV (6/60 events) less frequently than when using PCV (7/60 events). No significant difference in the number of hypercapnia occurrences between the VCV and PCV groups was detected (p=0.76) (Table-1).

Hypotension (mean arterial blood pressure <60 mmHg) was compared between the VCV and PCV groups. There was no significant difference in hypotension between the different tidal volume settings of the VCV group and the different PIP settings of the PCV group. However, of the 60 total trials, there was significantly more hypotension in the PCV group (9/60 events) than in the VCV group (0/60 events; p=0.003) (Table-2).

**Table 2:** Effects of ventilation on occurrence of hypotension in cats undergoing volume-controlled ventilation and pressure-controlled ventilation.

Volume-controlled ventilation			Pressure-controlled ventilation			p-value
	
Tidal volume (mL/kg)	Hypotension (<60 mmHg)		Pressure (mmHg)	Hypotension (<60 mmHg)		
	
	Positive	Negative		Positive	Negative	
6	0	12	4	3	9	0.217
8	0	12	5	2	10	0.478
10	0	12	6	3	9	0.217
12	0	12	7	0	12	1.000
14	0	12	8	1	11	1.000
Total	0	60	30	9	51	0.003

**Figure-3:**
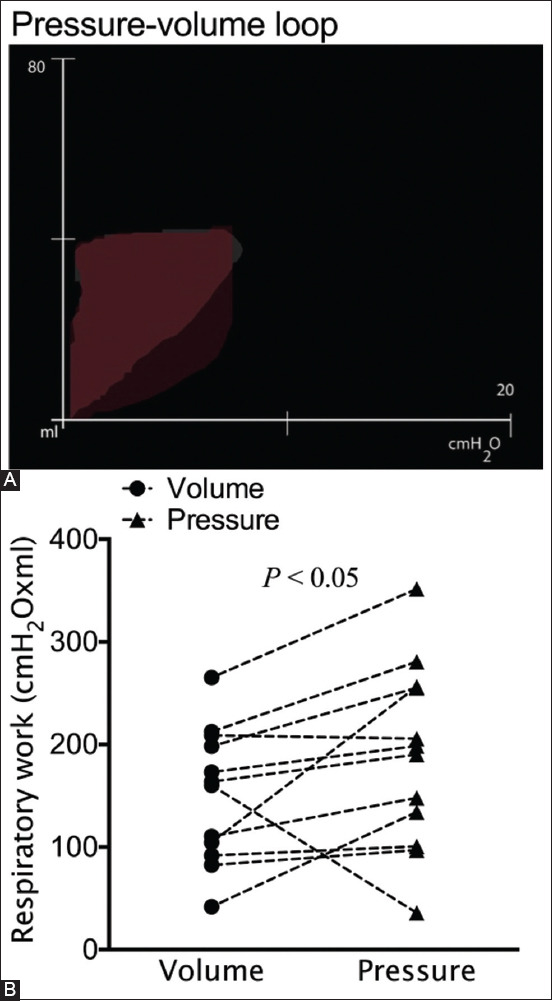
Overlaying pressure volume diagram in cats with a cuffed endotracheal tube ventilated with volume-controlled ventilation (VCV) and pressure-controlled ventilation (PCV) (A) and the work of breathing associated with VCV versus PCV (B).

Measuring the work of breathing is necessary to evaluate the status of patients during the use of mechanical controlled ventilation [19]. The work of breathing, the integral of the product of volume and pressure, was also compared between the VCV and PCV groups. The respiratory work in the PCV group was significantly higher than that in the VCV group (p<0.05; Figure-3). A higher respiratory work indicates that a greater amount of energy is required to overcome the elastic and resistive properties of the respiratory system.

## Discussion

The efficacy of ETT in cats under general anesthesia with VCV or PCV was evaluated in the present study. Our results revealed no difference in leakages when comparing VCV and PCV. Hypercapnia was less frequently identified when using VCV compared with PCV, but the frequency did not reach statistical significance. Hypotension occurred at a significantly lower frequency in the VCV group than in the PCV group. Moreover, VCV provided significantly lower work of breathing compared with PCV. Our results suggested that VCV not only provided a more stable tidal volume compared with PCV but also was associated with fewer complications.

Leakage during ventilation was evaluated in the present study. Our results indicated that there was no significant difference in respiratory leakage between VCV and PCV. Peak pressure below the cuff pressure and high-volume/low-pressure cuffs was used; therefore, there was no leakage in the present study. In a previous study, airway leakages were found less in the ETT group than in the supraglottic airway device group because the ETT provides a better air seal than supraglottic airway devices [1]. The cuff pressure of an ETT should be 20–30 cm H_2_O to minimize damage to tracheal mucosa, the increased risk of aspiration pneumonia, and interference with mechanical ventilation [1,7].

The volume of dead space is also influenced by the alteration of tidal volume and the frequency of ventilation [20]. In the present study, ETCO_2_ was monitored, and the dead space volume of the ETT and the minute ventilation in both groups were controlled. There was no significant difference in the presence of hypercapnia when comparing VCV and PCV. Nonetheless, a higher occurrence of hypotension in cats with PVC was identified compared with VCV. Positive pressure ventilation has both positive and negative hemodynamic effects [9]. The positive effects are improving gas exchange, decreasing the work of breathing, and resting the respiratory muscles. Ventilation may induce hemodynamic changes by altering systemic venous return [21]. When the lung volume changes and intrathoracic pressure is increased, there can be a reduction in systemic venous return to the heart and, at the same time, a decrease in afterload to the left ventricle and cardiac output [6,7,9,22].

The indications of mechanical ventilation for VCV and PCV are severe hypoxemia despite oxygen therapy (PaO_2_<60 mmHg), severe hypoventilation (PCO_2_>60 mmHg), severe circulatory shock, and excessive work of breathing [23]. The work of breathing is determined by the pressure-volume characteristics of the respiratory system. Work is needed to overcome the tendency of the lung to collapse. Our results revealed that VCV is associated with a lower work of breathing and a more stable tidal volume compared with PCV, resulting in a more stable minute volume. It should be noted that chronic use of a ventilator is associated with increased work of breathing and may lead to respiratory failure in humans. Thus, a mechanical ventilator with a lower work of breathing is preferred. Nonetheless, VCV should not be used in patients with an airway obstruction because a higher peak airway pressure may occur, leading to lung injury.

### Limitations of the study

One limitation of the present study is the small sample size. Because the study was designed using a paired sample, the use of a small sample size was feasible. Another limitation is the use of only healthy cats; the results of this study cannot be applied to cats with respiratory diseases. Mechanical ventilation is fraught with numerous adverse outcomes, such as airway complications, pneumothorax, interstitial emphysema, barotrauma, infection, and hypotension, in patients with pre-existing hypovolemia because positive end-expiratory pressure increases the intrathoracic pressure and reduces systemic venous return and cardiac output [24]. In addition, the present study only evaluated the short-term effects of mechanical ventilation using VCV and PCV. Therefore, more studies, especially those examining the long-term effects of mechanical ventilation, are required to further evaluate the effects of VCV and PCV in patients with prolonged use of mechanical ventilators.

## Conclusion

Our results indicate that VCV causes less hypotension than PCV. VCV also provides a more stable tidal volume compared with PCV, resulting in a more stable minute volume. Nonetheless, VCV should not be used in patients with an airway obstruction because a higher peak airway pressure may occur and lead to lung injury.

## Authors’ Contributions

NN: Designed the study, conducted literature review, performed the study, interpreted data, and drafted manuscript. NT: Designed the study, interpreted data, and reviewed the manuscript. All authors read and approved the final manuscript.
